# Considering Genetic Heterogeneity in the Association Analysis Finds Genes Associated With Nicotine Dependence

**DOI:** 10.3389/fgene.2019.00448

**Published:** 2019-05-17

**Authors:** Xuefen Zhang, Tongtong Lan, Tong Wang, Wei Xue, Xiaoran Tong, Tengfei Ma, Guifen Liu, Qing Lu

**Affiliations:** ^1^Department of Health Statistics, School of Public Health, Shanxi Medical University, Taiyuan, China; ^2^Department of Epidemiology and Biostatistics, Michigan State University, East Lansing, MI, United States

**Keywords:** nicotine dependence, genetic heterogeneity, *nAChRs* genes, SAGE, weighted U

## Abstract

While substantial progress has been made in finding genetic variants associated with nicotine dependence (ND), a large proportion of the genetic variants remain undiscovered. The current research focuses have shifted toward uncovering rare variants, gene-gene/gene-environment interactions, and structural variations predisposing to ND, the impact of genetic heterogeneity in ND has been nevertheless paid less attention. The study of genetic heterogeneity in ND not only could enhance the power of detecting genetic variants with heterogeneous effects in the population but also improve our understanding of genetic etiology of ND. As an initial step to understand genetic heterogeneity in ND, we applied a newly developed heterogeneity weighted U (HWU) method to 26 ND-related genes, investigating heterogeneous effects of these 26 genes in ND. We found no strong evidence of genetic heterogeneity in genes such as *CHRNA5*. However, results from our analysis suggest heterogeneous effects of *CHRNA6* and *CHRNB3* on nicotine dependence in males and females. Following the gene-based analysis, we further conduct a joint association analysis of two gene clusters, *CHRNA5*-*CHRNA3*-*CHRNB4* and *CHRNB3*-*CHRNA6*. While both *CHRNA5*-*CHRNA3*-*CHRNB4* and *CHRNB3*-*CHRNA6* clusters are significantly associated with ND, there is a much stronger association of *CHRNB3*-*CHRNA6* with ND when considering heterogeneous effects in gender (*p*-value = 2.11E-07).

## Introduction

Cigarette smoking is a leading cause of preventable disease, contributing to 5 million premature deaths worldwide each year. Nicotine dependence (ND, abbreviations are summarized in Supplementary Table S1) plays a critical role in smoking behavior since it is the dependence process that (a) drives up the count of occasions of tobacco use, (b) fosters escalation of dose, and (c) generates the multiple dose-years that account for the breadth of tobacco-attributable morbidity and mortality (e.g., cancers). Family, twin, and adoption studies have shown substantial genetic influence on ND (e.g., heritability of nicotine dependence is estimated from 50 to 60%). Substantial progress has also been made through linkage, candidate gene, and genome-wide association studies (GWAS) in identifying ND-associated genetic variants (Furberg et al., 2010; Sullivan et al., 2010; Wei et al., 2012).

Among all the findings, the neuronal nicotinic acetylcholine receptors (*nAChRs*) subunit genes have attracted special interest. *nAChRs* activates the release of dopamine, playing an important role in the dopaminergic reward system and the development of ND. *nAChRs* are pentameric complexes assembled from a family of subunits, encoded by 9 α and 3 β genes. Studies found strong associations of the *CHRNA5-CHRNA3-CHRNB4* cluster and the *CHRNB3-CHRNA6* cluster (Bierut et al., 2007; Saccone et al., 2007) with ND. Especially, a non-synonymous coding single nucleotide polymorphisms (SNP) in *CHRNA5*, rs16969968, has been identified and confirmed in several large-scale studies and meta-analysis (Liu et al., 2010; Hancock et al., 2015). Besides *nAChRs* subunit genes, cytochrome P450 (CYP) genes have also received increasing attention. *CYP* genes play a critical role in the synthesis and metabolism of various molecules and chemicals within cells. Studies have found the potential role of *CYP2A6* variants (e.g., *rs4105144*) in nicotine metabolism (Thorgeirsson et al., 2010).

Despite these achievements, the currently confirmed genetic variants only account for a small proportion of ND variation and a large proportion of ND loci remain undetected. While current efforts have been focused on rare variants and gene-gene interactions contributing to ND, little attention has been paid on the heterogeneous genetic etiology of ND. Traditionally, genetic heterogeneity refers to allelic/locus heterogeneity, i.e., different alleles or different loci lead to the same or similar phenotypes (Thornton-Wells et al., 2004). In the paper, genetic heterogeneity of ND is defined as a ND-associated genetic variant having different effects on individuals or on subgroups of a population (e.g., different gender groups). Compelling evidence from previous studies suggest that genetic etiology of ND could vary in subgroups (e.g., in males and females) (Li et al., 2003). When genetic heterogeneity is present, the current analysis will likely yield attenuated estimates of genetic effects, leading to low power of the study. Despite the strong evidence of heterogeneity in ND, investigating genetic variants with heterogeneous effects in ND remains a great challenge, due to issues such as multiple testing and reduced sample size in each subgroup.

To study the genetic heterogeneity of ND, we started with a gene-based association analysis of 26 ND-related genes, considering their heterogeneous effects in males and females. Following this analysis, we further investigated 2 *nAChRs* gene clusters, evaluating their heterogeneous effects in males and females. An advanced non-parametric method (Wei et al., 2016) has been implemented in this study to address issues of multiple testing and reduced sample size of subgroups, which facilitate our investigation of the genetic heterogeneity of ND.

## Materials and Methods

### Data Resource

Study of Addiction: Genetics and Environment (SAGE) is one of the largest and most comprehensive population-based studies conducted to date, aimed at discovering genetic contributions to addiction. Samples from the SAGE were selected from 3 large, complementary datasets: the Collaborative Study on the Genetics of Alcoholism (COGA), the Family Study of Cocaine Dependence (FSCD), and the Collaborative Genetic Study of Nicotine Dependence (COGEND). Multiple phenotypes were measured in the SAGE studies, including several nicotine dependence measurements (e.g., DSM IV ND). For the focus of our study, we use the Fagerstrom Test for Nicotine Dependence (FTND) item 4, the number of cigarettes smoked per day (CPD), which has been commonly used in genetic studies of nicotine use and dependence (Furberg et al., 2010). In SAGE, all subjects are classified into four categories based on participant’s lifetime score for CPD. The ordinal categorization is designed to examine the transition from nicotine use to dependence, ranging from very low dependence to very high dependence. The classes have been scored as very low (0), moderate (1), high (2) and very high (3). The SAGE studies’ assessment plans for personal characteristics (e.g., age) and for environmental conditions and processes (e.g., physical abuse) were also guided by standardized interview protocols and assessments, as described in the prior SAGE publications (Bierut et al., 2002, 2008). Table 1 summarizes demographic details of study samples (e.g., gender and ethnicity). SAGE comprises 1445 female and 1272 male samples, among which 807 samples are African-American and 1910 samples are Caucasian. The mean age from three studies ranges from 36.8 to 42.4.

**TABLE 1:** Distribution of the SAGE sample by gender, race and CPD.

		Study			Total sample
		COGA	COGEND	FSCD	
Gender	Male	534(59.47%)	442(35.00%)	296(53.24%)	1272(46.82%)
	Female	364(40.53%)	821(65.00%)	260(46.76%)	1445(53.18%)
Race	Caucasian	623(69.38%)	1019(80.68%)	268(48.20%)	1910(70.30%)
	African-American	275(30.62%)	244(19.32%)	288(51.80%)	807(s29.70%)
Mean age		42.4	36.8	37.0	38.7
CPD	0	256(28.51%)	941(74.51%)	156(28.06%)	1353(49.80%)
	1	387(43.09%)	177(14.01%)	225(40.47%)	789(29.04%)
	2	131(14.59%)	60(4.75%)	60(10.79%)	251(9.24%)
	3	124(13.81%)	85(6.73%)	115(20.68%)	324(11.92%)
Total sample		898	1263	556	2717

### Genotype and Quality Control

SAGE genotyping was performed at the Johns Hopkins University Center for Inherited Disease Research by using the Illumina Human 1M DNA Analysis BeadChip. The GENEVA Coordinating Center (CC) performed genotype imputation by using the BEAGLE software. Imputation was done separately for subjects with European and African ancestry, with references panels selected from HapMap Phase III populations. The minimum posterior probability required to call a genotype is 0.9. We merged the imputed SNPs with the genotyped SNPs, and then assembled multiple SNPs into 26 ND-related genes based on the Genome Reference Consortium release version 37 (GRCh37). Supplementary Table S2 in the Supplementary Material summarizes the 26 ND-related genes analyzed in this study. Prior to the statistical association analysis, we assessed the quality of the genotype data. As a first step in quality assessment, we examined the proportion of genotype calls for each marker (across all individuals) and for each individual (across all markers). Markers with less than 90% of successful calls were removed. Similarly, individuals with 10% missing genotypes were also excluded from the analysis. For the remaining missing genetic data, we used the average number of minor alleles of the marker to impute the missing values. Markers showing excessive deviations from Hardy-Weinberg equilibrium in the controls were marked and the individuals with unexpected relationships were removed.

### Heterogeneity Weighted U Method

Compelling evidence from previous studies suggest that genetic etiology of ND could vary in males and females (Li et al., 2003). To study the heterogeneous etiology of ND, we focused on 26 ND-related genes and investigated their potential roles in ND genetic heterogeneity. Despite the strong evidence of heterogeneity in ND, it is analytically challenging to investigate heterogeneous effects of genetic variants, primarily because the commonly used subset/stratified analysis is subject to issues of reduced sample size and multiple testing. Furthermore, the ND diagnostic assessments can often be of different types (e.g., binary and continuous phenotypes), with unknown underlying distributions (Neale et al., 2006). For example, the commonly used nicotine dependence measurement, CPD, do not follow a standard distribution (e.g., Poisson distribution). Most of the existing methods are parametric-based or semi-parametric-based, which often rely on certain assumptions (e.g., a Poisson distribution assumption). When the assumptions are violated, they are subject to power loss and/or false positive results (Wei et al., 2014).

To address these, we used our recently developed heterogeneity weighted U (HWU) method (Wei et al., 2016) to test the association of the 26 genes with ND considering genetic heterogeneity. The method is based on a weighted U statistic, which assume no specific distribution for phenotypes and can be applied to different types of ND phenotypes (e.g., binary, ordinal, and continuous phenotypes). It is also a similarity-based method, which can be applied to high-dimensional genetic data (i.e., the number of SNPs in a gene can be great than the study sample size). HWU is constructed based on the idea that the more similar subjects are, the more similar their genetic effects are (Wei et al., 2016). According to the idea, we defined HWU as,

$$U=2\,\sum_{1\leq i\leq j\leq n}w_{i,j}\,S_{i,j},$$

where *S_i,j_* is the phenotypic similarity between subjects i and j, and *w_i,__j_* is a weight function measuring the genetic similarity defined as $w_{i,j}=k_{i,j}\,f\,\left(G_{i},G_{j}\right)$ In this paper, we use cross-product kernel to measure the genetic similarity $f\,\left(G_{i},G_{j}\right)$ and use gender to infer the latent population structure by defining $k_{i,j}=1-\left|x_{i}-x_{j}\right|$, where $x_{i}=0,1$ for male and female, respectively. Under the null hypothesis, there is no association between phenotypic similarity and genetic similarity. Under the alternative, the phenotypic similarity increases with the increase of the genetic similarity. By constructing a background similarity on variables capturing heterogeneous groups (e.g., gender groups) and incorporating it into the weighted U framework, it can consider genetic heterogeneity in the association analysis. The method was implemented in a C++ software^1^. By applying the software to each of 26 candidate genes, we evaluated their association with ND with the consideration of possible heterogeneous effects in gender. To consider the potential confounding effects, we adjusted the analysis for gender, race, study sites and the top four principal components calculated from the genome-wide data. Because HWU is a non-parametric method, it only evaluates the significance of association but not measures of association. To measure the association, regression-based methods, such as zero inflated Poisson model, can be used.

## Results

### Gene-Based Association Analysis

Previous study showed that males and females differ in ND, which was also suggested by our own data (Table 2). Considering that the genetic etiology of ND could be heterogeneous in gender (Li et al., 2003), we applied the HWU method to 26 genes previously reported to be associated with ND, investigating potential genetic heterogeneity of these genes in males and females. We started the analysis with an overall association analysis considering potential genetic heterogeneous in gender (i.e., by incorporating the gender similarity into the association test).

**TABLE 2:** The distribution of CPD in males and females.

	Gender		
CPD	Male	Female	Total
0	484 (35.77%)	869 (64.23%)	1353
1	435 (55.13%)	354 (44.87%)	789
2	157 (62.55%)	94 (37.45%)	251
3	196 (60.49%0	128 (39.51%)	324
Total	1272 (46.82%)	1445 (53.18%)	2717
χ^2^ = 137.49, df = 3, *p* < 2.2e-16			

By considering genetic heterogeneity in gender, the analysis identified 24 genes significantly associated with CPD (Table 3). After Bonferroni correction, 17 genes remained statistically significant. The genes included 16 *CHRN* genes (including *ATR*1, *IREB2*, *MINK1*, and *PTK2B*), and 1 *CYP* genes. Four of the significant *CHRN* genes were on chromosome 8, 5 were on chromosome 15, 2 were on chromosome 17, and other six genes were located on different chromosomes locations. Comparatively, only one gene was significant after Bonferroni correction, while the genetic heterogeneity in gender was not considered. The detailed association results are summarized in Table 3. Additional analysis using FDR obtained similar results.

**TABLE 3:** Summary of ND-associated genes by HWU method.

Gene	Chrom	#SNPs	*P*_value_HG^1^	*P*_value_NHG^2^
*CHRNA6*	8	42	5.15E-05	0.297
*CHRNB3*	8	163	1.53E-04	0.011
*CHRNA7*	15	205	6.49E-04	0.402
*CHRNB2*	1	32	6.49E-04	0.153
*ART1*	11	55	6.81E-04	0.129
*CHRNA1*	2	60	0.004	0.952
*CHRNA9*	4	112	0.004	0.127
*CHRNE*	17	36	0.004	0.236
*CHRNB4*	15	24	0.009	0.127
*MINK1*	17	147	0.009	0.244
*CHRNA2*	8	96	0.012	0.504
*CHRNA5*	15	64	0.012	0.116
*CHRNA3*	15	69	0.013	0.116
*PTK2B*	8	392	0.013	0.123
*CHRNA4*	20	29	0.018	0.153
*IREB2*	15	128	0.029	0.131
*CYP2B6*	19	131	0.038	0.200
*LOC123688*	15	84	0.052	0.153
*CHRNG*	2	40	0.062	0.315
*FGF11*	17	28	0.073	0.360
*CHRND*	2	35	0.098	0.402
*PSMA4*	15	42	0.099	0.315
*CYP2A6*	19	14	0.115	0.135
*CHRNB1*	17	40	0.118	0.463
*ZBTB4*	17	83	0.274	0.402
*NUP98*	11	254	0.362	0.499

*${}^{1}$*P*_value_HG is the *P*-values considering genetic heterogeneity after Bonferroni correction. ${}^{2}$*P*_value_NHG is the *P*-values without considering genetic heterogeneity after Bonferroni correction.*

### Associated Analysis of Two ND-Related Gene Clusters

Following the single-gene analysis, we also conducted a joint association analysis of two gene clusters, *CHRNA*5-*CHRNA*3-*CHRNB*4 and *CHRNB*3-*CHRNA*6. The *CHRNA*5-*CHRNA*3-*CHRNB*4 cluster is on chromosome 15 and is of particular interest for its established association with ND. The association were identified and replicated in multiple studies (Saccone et al., 2007, 2009; Wen et al., 2016a). The *CHRNB*3-*CHRNA*6 cluster is on chromosome 8. The protein produced by the gene cluster are co-localized in *nAChRs* in the substantia nigra, ventral tegmental area, striatum, and locus coeruleus. While it has been less studied than the *CHRNA*5-*CHRNA*3-*CHRNB*4, studies have found SNPs in the *CHRNB3-CHRNA6* cluster are also significantly associated with ND (Culverhouse et al., 2014; Wen et al., 2016b).

The results of the joint analysis of the two gene clusters are summarized in Table 4. As expected, we found that the *CHRNA*5-*CHRNA*3-*CHRNB*4 cluster and *CHRNB*3-*CHRNA*6 cluster were significantly associated with ND in our study with or without considering gender heterogeneity. However, by considering gender heterogeneity, both gene clusters attained stronger associations with ND. The evidence of gender heterogeneity is particular strong in the *CHRNB*3-*CHRNA*6 cluster. Furthermore, the joint analysis of *CHRNB*3-*CHRNA*6 cluster attains a stronger association (*p*-value = 2.11E-07) than the single-gene analysis of *CHRNB*3 (*p*-value = 1.18E-05) and *CHRNA*6 (*p*-value = 1.98E-06).

**TABLE 4:** The association of two gene clusters with ND.

Gene clusters	*P*_value_HG^1^	*P*_value_NHG^2^
*CHRNA*5-*CHRNA*3-*CHRNB*4	4.59E-03	1.17E-02
*CHRNB*3-*CHRNA*6	2.11E-07	7.36E-04

*${}^{1}$*P*-values from a joint association analysis considering genetic heterogeneity. ${}^{2}$*P*-values from a joint association analysis without considering genetic heterogeneity.*

**FIGURE 1:**
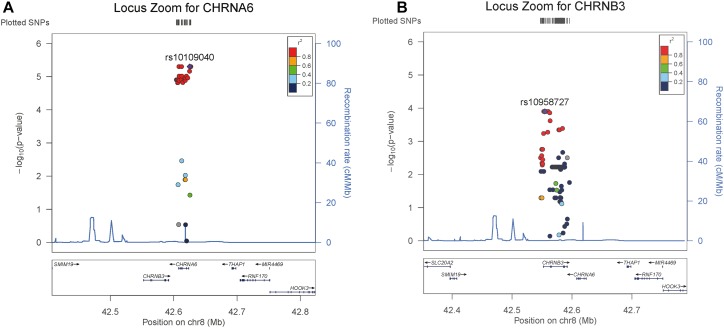
Summary of single-locus analysis of two interested genes by considering genetic heterogeneity; Panel **(A)** includes 6 SNPs within or near *CHRNA6* that are in complete linkage disequilibrium (LD) and are highly associated with CPD (*P*-value = 4.97E-06); Panel **(B)** includes five SNPs within or near *CHRNB3* that reach significance level of 1.25E-04 and are also in complete LD.

In order to further explore the association of the *CHRNB3*-*CHRNA6* gene cluster with ND, we performed a single-locus analysis of all SNPs located in these two gene clusters by using HWU. The results are summarized in Figure 1. From the single-locus analysis, we found six SNPs within or near *CHRNA6* were in complete linkage disequilibrium (LD) and were highly associated with CPD (*P*-value = 4.97E-06). In addition, 5 SNPs within or near *CHRNB3* reach significance level of 1.25E-04 and are also in complete LD. Additional results (e.g., coefficient estimates) from stratified analysis also suggest that there are heterogeneous effects of the two genes in males and females. For example, by using a zero inflated Poisson model, we found rs10109040 in *CHRNA6* has a coefficient estimate of −0.18 with a *p*-value of 2.47E-13 in males and a coefficients estimate of 0.026 with a *p*-value of 0.23 in females; rs10958727 in gene *CHRNB3* has a coefficient estimate of −0.18 with a *p*-value of 3.35E-14 in males and a coefficient estimate of −0.016 with a *p*-value of 0.45 in females.

## Discussion

In this study, we investigated the heterogeneous effects of 26 ND-associated genes in males and females. We found 17 genes significantly associated with ND by considering genetic heterogeneity in males and females. The most significant findings were from two *nAChRs* subunit genes, *CHRNA6* and *CHRNB3*, of which we found strong evidence of gender heterogeneity. The Human Protein Atlas^2^ shows that *CHRNB3* is expressed mostly in the brain, pancreas, and testis, and is a male-tissue-enriched gene (Fagerberg et al., 2014). On the other hand, *CHRNA6* is expressed specifically in the female tissues (e.g., the fallopian tube) and has almost no expression in male tissues. Our analysis indicated that variants in *CHRNA6* and *CHRNB3* (i.e., rs10109040 and rs10958727) had significant effects on ND in males but not in females. While the finding from *CHRNB3* is consistent with previous literature, the one from *CHRNA6* is not supported by previous findings. Significant association of *CHRNA6* with ND in males has also been reported in a recent study (Wen et al., 2017), which could be explained by the high linkage disequilibrium between *CHRNA6 and CHRNB3* (i.e., it may not be a real association signal).

Another group of interesting genes were from the *CHRNA5-CHRNA3-CHRNB4* cluster. Numerous studies suggested that the *CHRNA*5-*CHRNA*3-*CHRNB*4 genes cluster contributed to ND, which was also confirmed by our analysis. Several studies showed that the rare variants in *CHRNA3* and *CHRNB4* were associated with decreased risk for ND. Furthermore, the carriers of missenses variants in the *CHRNB4* were found to smoke less cigarettes per day (Haller et al., 2012; Wen et al., 2016a). The non-synonymous SNP rs16969968 is a well-known risk variant in *CHRNA5*, which has been replicated in many studies (Saccone et al., 2009; Wen et al., 2016a). While genes in the *CHRNA5-CHRNA3-CHRNB4* cluster are well-known for their role in ND, our study shows there is limited evidence of gender heterogeneity of these genes, which is consistent with findings from previous studies.

Biological studies showed that *CHRNA4* and *CHRNB2* could be related to ND (Marks et al., 1992; Whiteaker et al., 1998). Previous studies found that *CHRNA4* and *CHRNB2* were over-expressed under chronic nicotine exposure. Finding from another study also suggested that functional rare variants in *CHRNA4* might reduce ND risk (Xie et al., 2011). While existing literatures have been controversial on the association of *CHRNA4* and *CHRNB2* with ND, results from our association analysis supports the association of these two genes with ND.

*CHRNA1*, *CHRNA7*, and *CHRNB1* have been less studied for their role in ND. However, there is evidence that genetic variants in these genes contribute to ND (Philibert et al., 2009). Experiment studies reported that the *CHRNA*7 gene within the ventral tegmental area was directly involved in nicotine tolerance and withdrawal (Nomikos et al., 2000; Ryan and Loiacono, 2001; Grabus et al., 2005). Moreover, a study showed the association of *CHRN* genes (e.g., *CHRNA7*) with nicotine dependence differed in males and females (Philibert et al., 2009).

In this study, we also studied the relationship between nicotine dependence and two CYP genes, *CYP2A6* and *CYP2B6*. CYP genes encode the enzyme for the metabolism of xenobiotics in the body. Studies showed that *CYP2A6* influenced nicotine metabolism (Malaiyandi et al., 2005), while there was also evidence that this gene is not related with ND (Bandiera et al., 2015). Our finding is in line with the latter study. *CYP2B6* is associated with smoking behaviors and ND in human brain, and relapse could occur due to the rise of nicotine level in brain with *CYP2B6* activity dropping (Garcia et al., 2015).

The ND measurement, such as CPD, does not have a known underlying distribution. This complexity, however, has not been carefully considered in the existing analytic method. In our analysis, we adopted a new non-parametric method, HWU, which made no assumptions on phenotype distribution and therefore provided a robust and powerful performance for association analysis. Moreover, in HWU, we integrated the latent population structure (e.g., inferred from gender) into a weight function and test heterogeneous effects without stratifying the sample, which addressed the issues of reduced sample size and multiple testing. By using the new method, we were able to identify genes, such as *CHRNA6* and *CHRNB3*, associated with ND. To show the evidence of gender heterogeneity, we also performed an analysis without considering the heterogeneous effects. We found that most genes were not significantly associated with ND in this additional analysis, which could suggest the importance of considering gender heterogeneity. If the genetic mechanisms differ in male and female, performing the analysis without considering genetic heterogeneity will likely lead to low power of the study.

In summary, our findings are consistent with existing biological lectures or previous association analysis. While this study reveals potential heterogeneous effects of several ND-associated genes in gender, this is an initial effort to study genetic heterogeneity in ND. Future studies are required to replicate the findings from our analysis and further investigate genetic heterogeneity in ND. Because HWU is a non-parametric method, it is not straightforward to incorporate a variable selection algorithm into HWU. Alternatively, we could screen SNPs based on information theory, such as using an diSNP pre-selection method (Cen et al., 2012; Wu and Cui, 2014), which could potentially improve the power of association analysis. In addition to HWU, other high-dimensional statistical methods, especially robust variable selection methods considering genetic heterogeneity (Wu and Ma, 2015), can be adopted for association analysis considering genetic heterogeneity.

## Author Contributions

QL directed the study and contributed to the data interpretation. XT conducted the quality control process and modified the HWU method. XZ and TL performed the analysis. XZ and TM were involved in the result interpretation. XZ drafted the manuscript. TW, WX, and GL provided the suggestions and revised the manuscript. All authors reviewed and improved the submitted manuscript.

## Conflict of Interest Statement

The authors declare that the research was conducted in the absence of any commercial or financial relationships that could be construed as a potential conflict of interest.

## Footnotes

1 http://changshuaiwei.github.io/software.html#HWU

2 https://www.proteinatlas.org
