# Supplementary material for: Considering Genetic Heterogeneity in the Association Analysis Finds Genes Associated With Nicotine Dependence
Source: Front Genet. 2019 May 17;10:448. doi: 10.3389/fgene.2019.00448 (PMC6534062; doi:10.3389/fgene.2019.00448)
Supplement: Supplementary file 1 [file Table_1.DOCX]

Supplementary Material

Considering Genetic Heterogeneity in the Association Analysis Finds Genes Associated with Nicotine Dependence

Xuefen Zhang^1,2^, Tongtong Lan^1^, Guifen Liu^1^, Xiaoran Tong^2^, Tengfei Ma^2^, Tong Wang^1^ and Qing Lu^2^*

*** Correspondence:** Qing Lu: qlu@epi.msu.edu

# Supplementary table

Table S1 A list of abbreviations

| **Abbreviation** | **Nomenclature** |
| --- | --- |
| ND | Nicotine Dependence |
| SAGE | The Study of Addiction: Genetics and Environment |
| CC | The GENEVA Coordinating Center |
| COGA | The Genetics of Alcoholism |
| FSCD | The Family Study of Cocaine Dependence |
| COGEND | The Collaborative Genetic Study of Nicotine Dependence |
| FTND | Fagerstrom Test for Nicotine Dependence |
| CPD | The number of cigarettes smoked per day |
| GRCh37 | Genome Reference Consortium release version 37 |
| HWU | Heterogeneity Weighted U Method |
| LD | Linkage Disequilibrium |

Table S2 Summary of genes used in the analysis

| **Gene** | **Chromosome** | **Number of imputed SNPs** | **Number of observed SNPs** | **Total Number of SNPs** |
| --- | --- | --- | --- | --- |
| *ART1* | 11 | 43 | 12 | 55 |
| *CHRNA1* | 2 | 48 | 12 | 60 |
| *CHRNA2* | 8 | 68 | 28 | 96 |
| *CHRNA3* | 15 | 49 | 20 | 69 |
| *CHRNA4* | 20 | 21 | 8 | 29 |
| *CHRNA5* | 15 | 50 | 14 | 64 |
| *CHRNA6* | 8 | 36 | 6 | 42 |
| *CHRNA7* | 15 | 167 | 38 | 205 |
| *CHRNA9* | 4 | 98 | 14 | 112 |
| *CHRNB1* | 17 | 24 | 16 | 40 |
| *CHRNB2* | 1 | 20 | 12 | 32 |
| *CHRNB3* | 8 | 142 | 21 | 163 |
| *CHRNB4* | 15 | 16 | 8 | 24 |
| *CHRND* | 2 | 27 | 8 | 35 |
| *CHRNE* | 17 | 27 | 9 | 36 |
| *CHRNG* | 2 | 28 | 12 | 40 |
| *FGF11* | 17 | 17 | 11 | 28 |
| *IREB2* | 15 | 103 | 25 | 128 |
| *LOC123688* | 15 | 93 | 42 | 135 |
| *MINK1* | 17 | 118 | 29 | 147 |
| *NUP98* | 11 | 214 | 40 | 254 |
| *PSMA4* | 15 | 33 | 9 | 42 |
| *PTK2B* | 8 | 306 | 86 | 392 |
| *ZBTB4* | 17 | 69 | 14 | 83 |
| *CYP2A6* | 19 | 11 | 1 | 12 |
| *CYP2B6* | 19 | 99 | 30 | 129 |
| **Sum** |  | **1790** | **488** | **2278** |
